# Generalized contrast-to-noise ratio applied to short-lag spatial coherence ultrasound differentiates breast cysts from solid masses

**DOI:** 10.1093/radadv/umaf037

**Published:** 2025-10-24

**Authors:** Arunima Sharma, Eniola T Oluyemi, Madhavi Tripathi, Emily B Ambinder, Lisa A Mullen, Babita Panigrahi, Joanna Rossi, Nethra Venkatayogi, Kelly S Myers, Muyinatu A Lediju Bell

**Affiliations:** Department of Electrical and Computer Engineering, Johns Hopkins University, Baltimore, MD 21218, United States; Department of Radiology and Radiological Science, Johns Hopkins Medicine, Baltimore, MD 21287, United States; Department of Electrical and Computer Engineering, Johns Hopkins University, Baltimore, MD 21218, United States; Department of Radiology and Radiological Science, Johns Hopkins Medicine, Baltimore, MD 21287, United States; Department of Oncology, Johns Hopkins Medicine, Baltimore, MD 21287, United States; Department of Radiology and Radiological Science, Johns Hopkins Medicine, Baltimore, MD 21287, United States; Department of Radiology and Radiological Science, Johns Hopkins Medicine, Baltimore, MD 21287, United States; Department of Radiology and Radiological Science, Johns Hopkins Medicine, Baltimore, MD 21287, United States; Department of Computer Science, Johns Hopkins University, Baltimore, MD 21218, United States; Department of Radiology and Radiological Science, Johns Hopkins Medicine, Baltimore, MD 21287, United States; Department of Electrical and Computer Engineering, Johns Hopkins University, Baltimore, MD 21218, United States; Department of Oncology, Johns Hopkins Medicine, Baltimore, MD 21287, United States; Department of Computer Science, Johns Hopkins University, Baltimore, MD 21218, United States; Department of Biomedical Engineering, Johns Hopkins University, Baltimore, MD 21218, United States

**Keywords:** breast ultrasound, complicated cysts, coherence-based beamforming, acoustic clutter reduction, reader assessment

## Abstract

**Background:**

Benefits of ultrasound in breast cancer detection are often limited by the similar appearance of complicated cysts and solid hypoechoic masses with B-mode imaging, which can lead to false positive diagnoses.

**Purpose:**

To evaluate the diagnostic performance of generalized contrast-to-noise ratio (gCNR) on short-lag spatial coherence (SLSC) ultrasound images as an objective tool to improve complicated cyst vs solid mass classification.

**Materials and Methods:**

For this secondary analysis of a prospective recruitment for the Advanced Ultrasound Signal Processing of Suspicious Breast Images (AUSPICIOUS) observational study (NCT07206888), women scheduled for ultrasound-guided procedures or follow-up of at least 1 breast mass were enrolled from March 2018 to October 2023. Raw ultrasound data were acquired with an Alpinion ECUBE12R research scanner, then post-processed with our custom software. The primary evaluation indicator was gCNR applied to SLSC images with regions of interest determined by 6 radiologists. Outcomes were compared to the same radiologists classifying mass contents as solid, fluid, mixed, or uncertain using B-mode images. The reference standard was determined by aspiration, pathology, or characterization of image features. Areas under receiver operating characteristic curves (AUCs) with 95% confidence intervals (CIs) and inter-reader agreement (Fleiss’ *κ*) were assessed.

**Results:**

Among 175 cases, 145 breast masses from 115 women (age: 52 ± 17 years) were analyzed, including 16 complicated cysts and 96 solid masses. The mean AUC for complicated cyst vs solid mass characterization was 0.96 (95% CI: 0.94, 0.97) with gCNR applied to SLSC images, relative to a mean lower-bound AUC of 0.67 (range: 0.54-0.76) with readings of B-mode images (*P* < .05). Inter-reader agreement improved from fair with B-mode (κ  =  0.40) to moderate with gCNR applied to SLSC images with a 0.76 threshold (κ  =  0.59, *P* < .00001).

**Conclusion:**

Applying an objective gCNR metric to SLSC images improved the differentiation of complicated cysts from solid masses when compared to subjective readings of B-mode images.

**Abbreviations** 

AUC = area under receiver operating characteristic curve; CI = confidence interval; gCNR = generalized contrast-to-noise ratio; ROC = receiver operating characteristic; ROI = region of interest; SLSC = short-lag spatial coherence; THI = tissue harmonic imaging

**Summary** 

The generalized contrast-to-noise ratio (gCNR), when applied to short-lag spatial coherence (SLSC) ultrasound images, outperformed radiologist interpretations of B-mode and SLSC images to differentiate cysts from solid masses.

**Key Results** 

- Complicated cysts initially categorized as BI-RADS 3 or 4 were identified as fluid with gCNR applied to SLSC images.
- The mean AUC among 6 readers differentiating complicated cysts from solid masses increased from 0.67 with qualitative B-mode to 0.96 with gCNR applied to SLSC (*P*<.05).
- Inter-reader agreement was fair with B-mode (κ = 0.40), moderate with B-mode+SLSC (κ = 0.41), and moderate with gCNR applied to SLSC (κ = 0.59).

## Introduction

Early detection of breast cancer reduces mortality.^1–3^ Ultrasound plays an important role in supplemental breast cancer screening in women with dense breast tissue and in the diagnostic evaluation of mammography findings or clinical symptoms. However, high false positive rates can lead to unnecessary follow-up exams and procedures, particularly for complicated cysts, as there can be overlap with the sonographic appearance of hypoechoic solid masses due to acoustic clutter.^4^

Tissue harmonic imaging (THI) can reduce acoustic clutter by incorporating nonlinear sound propagation.^5^^,^^6^ While THI improves grayscale contrast between fatty tissue and breast lesions, which may lead to improved lesion detectability and characterization,^7^^,^^8^ this improvement is greater with fatty rather than dense breast tissue.^9^ Short-lag spatial coherence (SLSC) is an alternative technique, implemented as a prototype, to reduce acoustic clutter and improve ultrasound image quality,^10–14^ particularly in the presence of dense breast tissue.^15^ While conventional B-mode imaging and THI form images based on echo amplitude, SLSC images are based on local measurements of spatial coherence of backscattered echoes.^16^ The generalized contrast-to-noise ratio (gCNR), when applied to SLSC images, has sensitivity and specificity to classify cystic vs solid mass contents.^17^^,^^18^ SLSC is also superior to THI for this task.^17^ Preliminary SLSC breast imaging studies^16–18^ are limited to 8-44 patients and up to 60 masses, and none reference gCNR outcomes with assessments from the same readers.

The study herein aimed to evaluate the performance of gCNR applied to SLSC images in differentiating complicated cysts from solid masses in a larger patient cohort than that presented in preliminary studies.^16–18^ Diagnostic accuracy and inter-reader agreement were evaluated.

## Materials and methods

### Study population

Patients (132 total) scheduled for ultrasound-guided core-needle biopsy, aspiration, or follow-up of at least 1 breast mass were recruited and enrolled with inclusion criteria: adult female (>18 years), English speaker, and not pregnant. Except for recruitment being stalled during the COVID-19 pandemic and radiologist or researcher unavailability to operate the ultrasound scanner used in our study, consecutive cases were recruited. Table 1 summarizes the age of recruited participants. Prospective recruitment for secondary analysis of data acquired for the Advanced Ultrasound Signal Processing of Suspicious Breast Images (AUSPICIOUS) observational study (NCT07206888) was approved by the Johns Hopkins Medicine Institutional Review Board (Protocol No. IRB00127110), with written informed consent obtained from participants from March 4, 2018, through October 5, 2023.

**Table 1.** Demographics of study participants and mass characteristics.

Category	# of participants (% of total eligible)	Mean age ± standard deviation [years]	# of masses per category
Enrolled	132	52 ±17	175
Eligible for analysis	115	52 ±17	145
Mean lesion size ± standard deviation = 13.8 ± 9.8 mm	115	52 ±17	145
Mean lesion depth ± standard deviation = 1.1 ± 0.4 cm	115	52 ±17	145
BI-RADS category prior to enrollment			
BI-RADS 2	10/115 (9%)	56±13	11
BI-RADS 3	1/115 (1%)	48±0	1
BI-RADS 4	112/115 (97%)	52±17	131
BI-RADS 5	2/115 (2%)	58±5	2
Number of masses per participant			
1	90/115 (78%)	52±16	90
2	21/115 (18%)	50±19	42
3	3/115 (3%)	70±6	9
4	1/115 (1%)	45±0	4
Reference standard determined by			
B-mode image features	10/115 (9%)	56±13	11
Successful aspiration	13/115 (11%)	62±12	14
Core-needle biopsy and pathology	101/115 (88%)	51±17	119
Pathology from mass excision	10/101 (10%)	52±17	10
>2-year follow-up	1/115 (1%)	48±0	1
Masses included in statistical analyses			
Complicated cysts	15/115 (13%)	61±12	16
Solid masses	82/115 (71%)	51±17	96
Malignant solid	22/82 (27%)	65±12	25
Benign solid	62/82 (76%)	46±16	71
Histopathology of biopsied/excised masses			
Malignant			
Invasive ductal carcinoma	15/101 (16%)	65±14	18
Ductal carcinoma in situ	4/101 (4%)	62±7	4
Papillary carcinoma	2/101 (2%)	61±4	2
Infiltrating lobular carcinoma	1/101 (1%)	68±0	1
Infiltrating mammary carcinoma	1/101 (1%)	73±0	1
Benign			
Fibroadenoma	32/101 (32%)	38±12	38
Fibrocystic changes	12/101 (12%)	50±15	13
Stromal fibrosis	5/101 (5%)	62±19	6
Fat necrosis	4/101 (4%)	61±5	4
Fibroadenomatoid changes	2/101 (2%)	43±11	2
Pseudoangiomatous stromal hyperplasia	2/101 (2%)	58±18	2
Necrotizing granulomas consistent with abscess	1/101 (1%)	36±0	2
Other (less than 1% of masses)	25/101 (26%)	53±16	25
Race^a^			
Asian	4/115 (3%)	45±15	4
Black	53/115 (46%)	52±17	62
White	50/115 (43%)	53±15	70
Other/Unknown	8/115 (7%)	48±19	9
Ethnicity^a^			
Hispanic or Latino	3/115 (3%)	36±6	4
Not Hispanic or Latino	108/115 (94%)	52±17	137
Unknown	4/115 (3%)	57±17	4

a The source of the race and ethnicity categories is electronic health records, assessed as required by the U.S. National Institutes of Health (NIH), consistent with the Inclusion of Women, Minorities, and Children policy. Individuals participating in the study survey were categorized as American Indian or Alaska Native, Asian, Black or African American, Hispanic or Latino, Native Hawaiian or Other Pacific Islander, or White based on the NIH Policy on Reporting Race and Ethnicity Data.

A total of 175 masses were potentially eligible, with 30 masses excluded based on the following physical, pathological, or imaging characteristics: hyperechoic content (*n* = 2), unknown contents due to no definitive cyst characterization of BI-RADS 3 masses at the time of imaging (*n* = 16), incomplete follow-up for masses biopsied with high-risk pathology result (*n *= 2), no clinical screenshots available (*n* = 2), shallow and therefore not suitable for SLSC imaging^17^ (*n* = 4), or not completely visible in the 25.4 mm-wide ultrasound image field of view (*n* = 4). Of the 145 eligible masses included in the study, 128 (from 100 patients) were previously reported,^15–19^ including 6 (from 6 patients),^16^ 23 (from 22 patients),^19^ 29 (from 25 patients),^15^ 36 (from 28 patients),^17^ and 60 (from 44 patients),^18^ with additional mass and patient overlap among previous reports.

### Image acquisition and postprocessing

Raw radiofrequency ultrasound channel data were acquired with an Alpinion (Seoul, South Korea) ECUBE12R scanner, SDK software v1.2, and an Alpinion L8-17 probe (142 eligible masses) or Alpinion L3-8 probe (3 eligible masses). The SLSC processing code for this research ultrasound scanner is a prototype, which is not currently commercially available, but a publicly available implementation can be accessed through the Ultrasound Toolbox.^20^^,^^21^ Raw data from 2 orthogonal views of each mass were acquired by 1 of 3 board-certified breast radiologists (co-authors E.T.O., K.S.M., or E.B.A.) who operated the ultrasound probe, while a study team member operated the control panel of the scanner. Following the acquisition, raw data were processed with MATLAB 2023b to form B-mode and SLSC images. Two static orthogonal B-mode images of each mass were additionally acquired. Doppler imaging was not performed.

### Image analysis

Images of included breast masses were randomized and read independently by 6 board-certified breast radiologists, who were blinded to the reference standard (co-authors E.T.O., K.S.M., E.B.A., L.A.M., B.P., and J.R.). Each reader had experience beyond residency ranging from 5 to 27 years. Three readers (E.T.O., K.S.M., E.B.A.) assisted with image acquisition, and 4 readers (E.T.O., K.S.M., E.B.A., L.A.M.) had prior experience reading SLSC images.^19^ The remaining readers had no prior knowledge of enrolled patients, no prior experience with the SLSC imaging, and no participation in patient recruitment. However, each radiologist received a tutorial on SLSC image interpretation (Supplementary Material) prior to readings, which were performed in a dark room, with reader autonomy to manage pace and breaks to prevent fatigue.

To perform the readings, a graphical user interface^19^ presented Task 1 (evaluate B-mode images), then Task 2 (evaluate B-mode with corresponding SLSC images), then prompted the reader to advance to the next mass, until all masses in the study were read. Readers evaluated the content (ie, solid, fluid, mixed, or uncertain) and BI-RADS category per task per mass, after toggling between radial and antiradial views. Task 3 was then performed with each reader instructed to draw regions of interest (ROIs) that were used to apply gCNR^22^ to B-mode and SLSC images, which can be implemented using gCNR code provided through the Ultrasound Toolbox.^21^^,^^23^ Mass ROI drawing rules were designed to avoid boundaries in B-mode images and place the tissue ROI (automatically set to the same size) at the same depth in the ultrasound image. Areas of the ROIs ranged from 0.4 mm^2^ for the smallest mass (ie, 4 mm) to 100.5 mm^2^ for the largest mass (ie, 53 mm). BI-RADS 3 masses with unknown contents (included in Tasks 1 and 2) were excluded from Task 3. The same clinical history was presented per mass: “40 yo female with a baseline ultrasound exam.” When applying the ROIs to calculate the gCNR of SLSC images, the short-lag value was fixed to *M* = 7, corresponding to 10% of the receive aperture, using the full linear scale after truncating negative values.^17^

### Reference standard

Final diagnosis was determined by B-mode image features of simple cysts (*n* = 11), successful aspiration (*n* = 14), pathology following core-needle biopsy (*n* = 119) or surgical excision (*n* = 10), or characterization as a cyst during follow-up of BI-RADS 3 masses (*n* = 1). Simple cysts, complicated cysts, mixed echogenicity masses (eg, clustered microcysts, complex cystic and solid masses), benign solid masses, high-risk solid masses, and malignant solid masses were included in data analysis. To confirm complicated cysts, ultrasound-guided aspiration was initially attempted with successful aspiration indicating a complicated cyst. If attempted aspiration of a questioned cyst was unsuccessful, then the finding was classified as a solid mass and subsequently biopsied. One complicated cyst resolved by the time of the biopsy, and the surrounding tissue was biopsied and determined to be benign, enabling classification of the initial finding as a complicated cyst. One BI-RADS 3 mass was ultimately stable for >2 years and anechoic at final follow-up, confirming correct categorization as a complicated cyst at the time of imaging. The follow-up duration for this mass was 33 months. Otherwise, the pathology results of each core-needle biopsy (or pathology after excision of high-risk masses) served as the ground truth for mass classification.

### Statistical methods

Sensitivity and specificity for fluid mass classification were calculated for a subgroup of 112 masses (ie, 16 complicated cysts, 96 solid masses) to create receiver operating characteristic (ROC) curves. A true positive (TP) or false negative (FN) was a fluid mass above or below varied gCNR thresholds, respectively, and a true negative (TN) or false positive (FP) was a solid mass below or above the same gCNR thresholds, respectively. Masses with complex cystic and solid content (classified here as mixed) were excluded from statistical analyses because they contain both TP and TN. Simple cysts were additionally excluded because they can be classified as benign based on clinical B-mode ultrasound features consistent with a simple cyst. The areas under the ROC curve (AUCs) were estimated using trapezoidal numerical integration. Bootstrap resampling of gCNR values and associated classifications (1000 iterations) were used to determine 95% confidence intervals (CIs) for the mean AUC among the 6 radiologists.

A previous publication demonstrated thresholds ranging from 0.62 to 0.85 as potentially acceptable.^17^ Within this range, a threshold of 0.76 was chosen to optimize performance for a radiologist with prior experience reading SLSC images by maximizing the number of cysts correctly identified and minimizing the number of cancers misclassified as cysts. This threshold was then used to assess the distribution of gCNR values obtained for the 145 eligible masses (stratified by mass category), to assess example images, and to compare inter-reader agreement.

As a control for comparison, the sensitivity and 1-specificity of the subjective reader assessments were plotted on the same ROC curves noted above. The fraction of cysts correctly identified as fluid determined sensitivity, and the fraction of solid masses correctly identified as solid determined specificity. The lower-bound AUC was calculated as the integral from (0,0) to the plotted point for each reader to (1,1) on the ROC curve.^24^ Bootstrap resampling of 6 plotted points (1000 iterations, allowing duplicates) and the resulting lower-bound ROC curves were used to determine 95% CIs for the mean lower-bound AUC among the 6 readers.

The statistical methods described above were implemented using MATLAB 2025b (co-authors AS and MT). DeLong’s test was used to determine the statistical significance of AUC differences, implemented with MATLAB 2024b (co-author NV).^25^ Our intended sample size was at least 73 masses, determined using standard methods^26^^,^^27^ (ie, a 2-sided Z-test with a significance level of 0.05), which indicated that an AUC difference of 0.175 is detectable with 80% power with >11 complicated cysts and >62 solid masses (PASS 2025, version 25.0.1).

Fleiss’ *κ* was used to determine inter-reader agreement among the 6 radiologists,^28^ implemented with MATLAB 2025b (co-author MT). Values of *κ *< 0.0, 0-0.20, 0.21-0.40, 0.41-0.60, and 0.61-0.80, and >0.81 were interpreted as poor, slight, fair, moderate, substantial, and perfect agreement, respectively.^29^ Differences among values were assessed for statistical significance with the null hypothesis *κ *= 0, assuming a large-sample^30^ normal distribution, then calculating variance,^30^^,^^31^ standard error,^30^ and z-scores,^30^ and converting z-scores to *P* values at the .05 significance level.^32^

## Results

### Classifying breast cysts with SLSC and gCNR

**Figure 1.**
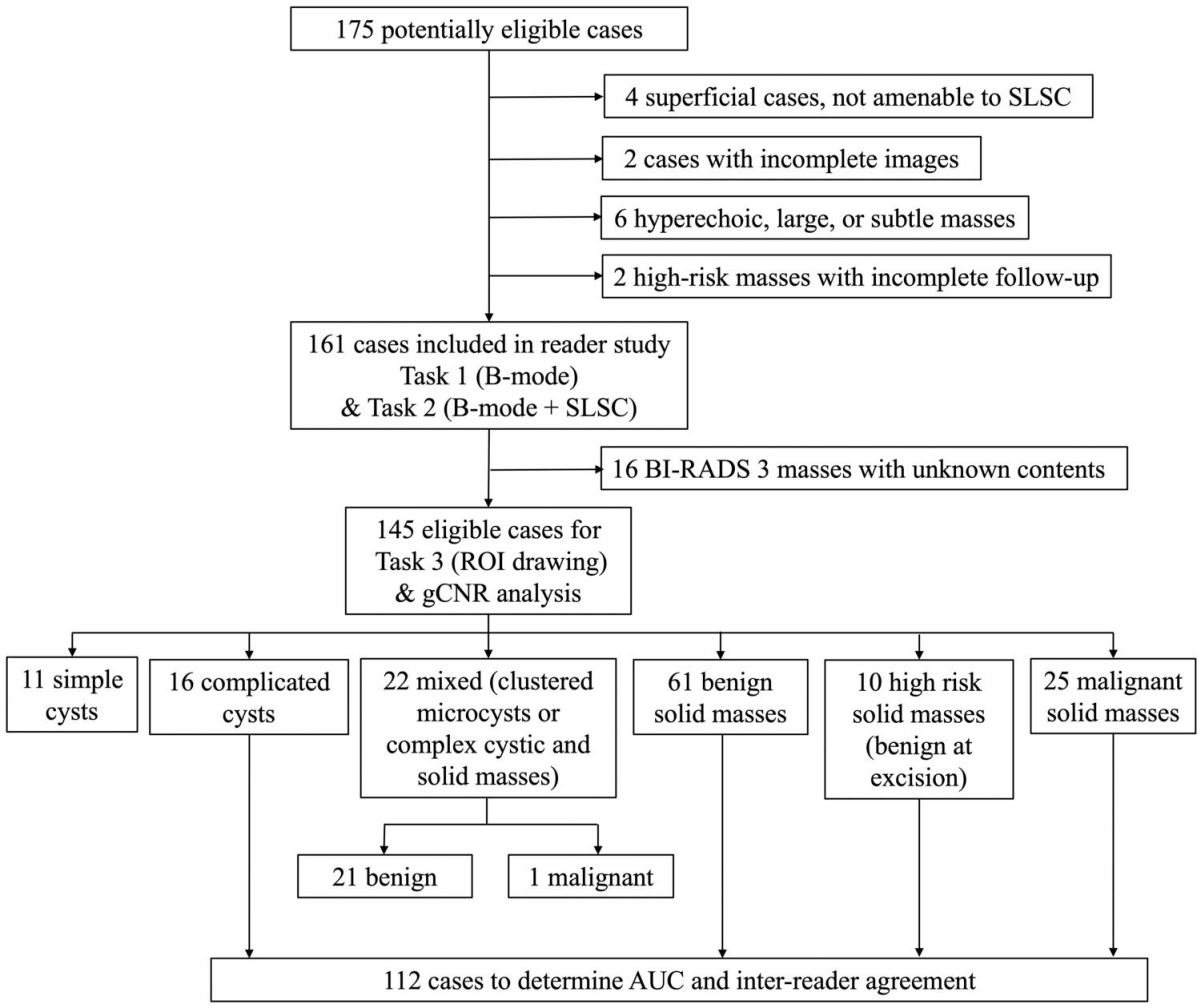
Flowchart of 145 breast masses (from 115 patients) eligible for analyzing the performance of 6 board-certified radiologists. Superficial cases are defined as shallow masses located <5 mm from the transducer, where it is difficult to focus ultrasound as required for successful short-lag spatial coherence (SLSC) imaging.^17^ Task 1: select mass content and BI-RADS category based on B-mode images; Task 2: select mass content and BI-RADS category based on B-mode + SLSC images; Task 3: draw regions of interest (ROIs) to calculate generalized contrast-to-noise ratios (gCNRs) on B-mode and SLSC images.

Of 175 potentially eligible sonographic masses, 145 masses in 115 patients were eligible and included in the application of gCNR to SLSC images, based on Task 3 of the reader study, as documented in Figure 1. Ninety patients had 1 mass, 21 patients had 2 masses, 3 patients had 3 masses, and 1 patient had 4 masses. The mean age was 52 years (range: 20 to 91). Most masses (96/145, 66%) were solid based on pathology, with 16/145 (11%) representing complicated cysts based on successful aspiration, pathology, or 2-year follow-up, 11/145 (8%) representing simple cysts based on B-mode image features, and 22/145 (15%) representing masses with mixed solid and cystic contents based on pathology. Prior to enrollment in our study, 8% (11/145), 0.7% (1/145), 90% (131/145), and 1.4% (2/145) of included masses were diagnosed as BI-RADS categories 2, 3, 4, and 5, respectively. The 11 simple cysts were categorized as BI-RADS 2, which supports the rationale for excluding simple cysts from the statistical analyses. The single BI-RADS 3 mass was determined to be a complicated cyst based on its appearance during 33 months of follow-up, while 14 complicated cysts were classified based on successful aspiration, and 1 was classified as a complicated cyst during biopsy. Of the 112 masses included in the statistical analyses, 16 were complicated cysts and 96 were solid masses, of which 71 were benign and 25 were malignant, as summarized in Table 1.

**Figure 2.**
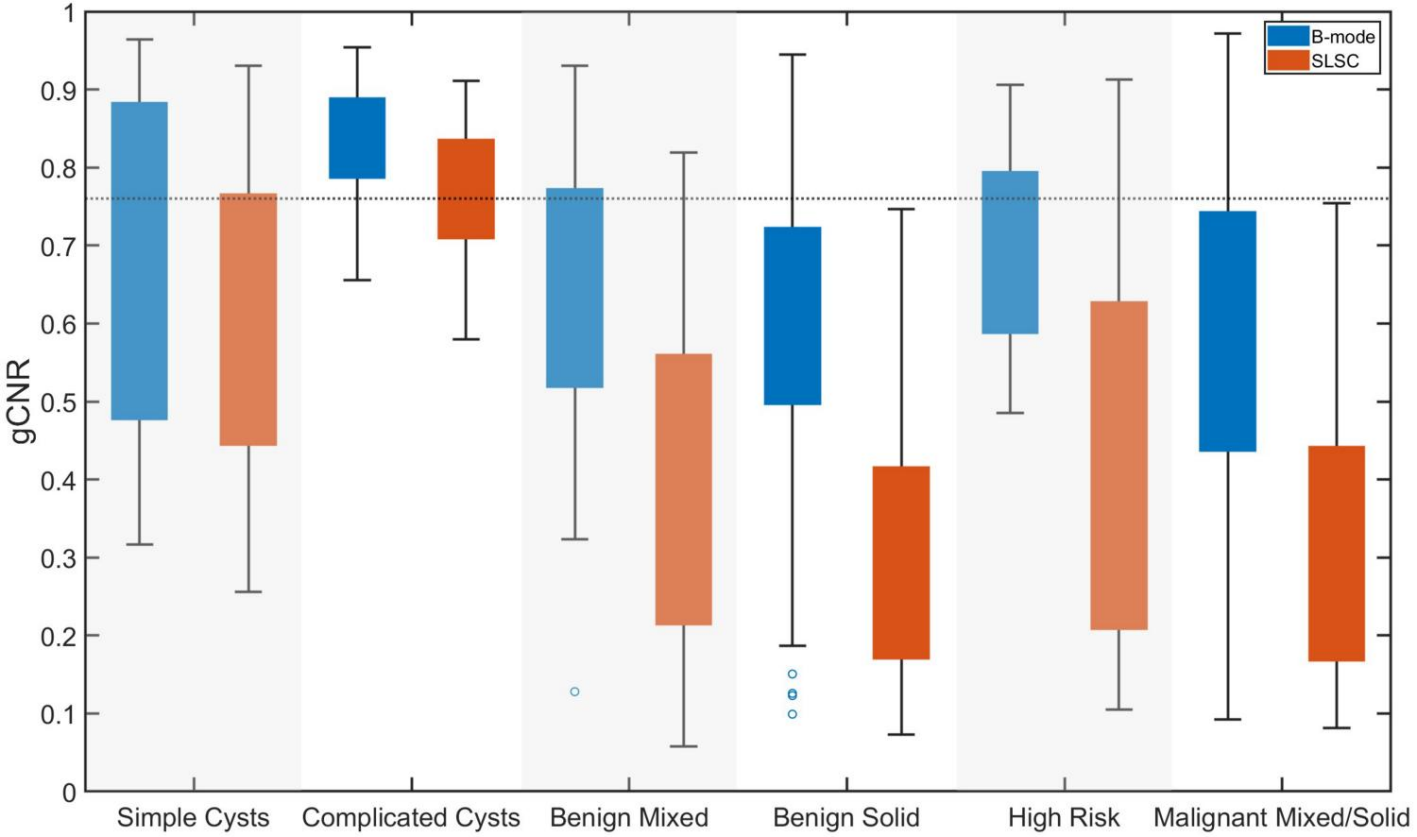
Box plot of generalized contrast-to-noise ratio (gCNR), when applied to B-mode and short-lag spatial coherence (SLSC) ultrasound images of 145 sonographic masses, categorized by internal contents and benign/malignant status. The 0.76 threshold was chosen to optimize performance within acceptable values ranging from 0.62 to 0.85 (based on prior research on this topic^17^).

**Figure 3.**
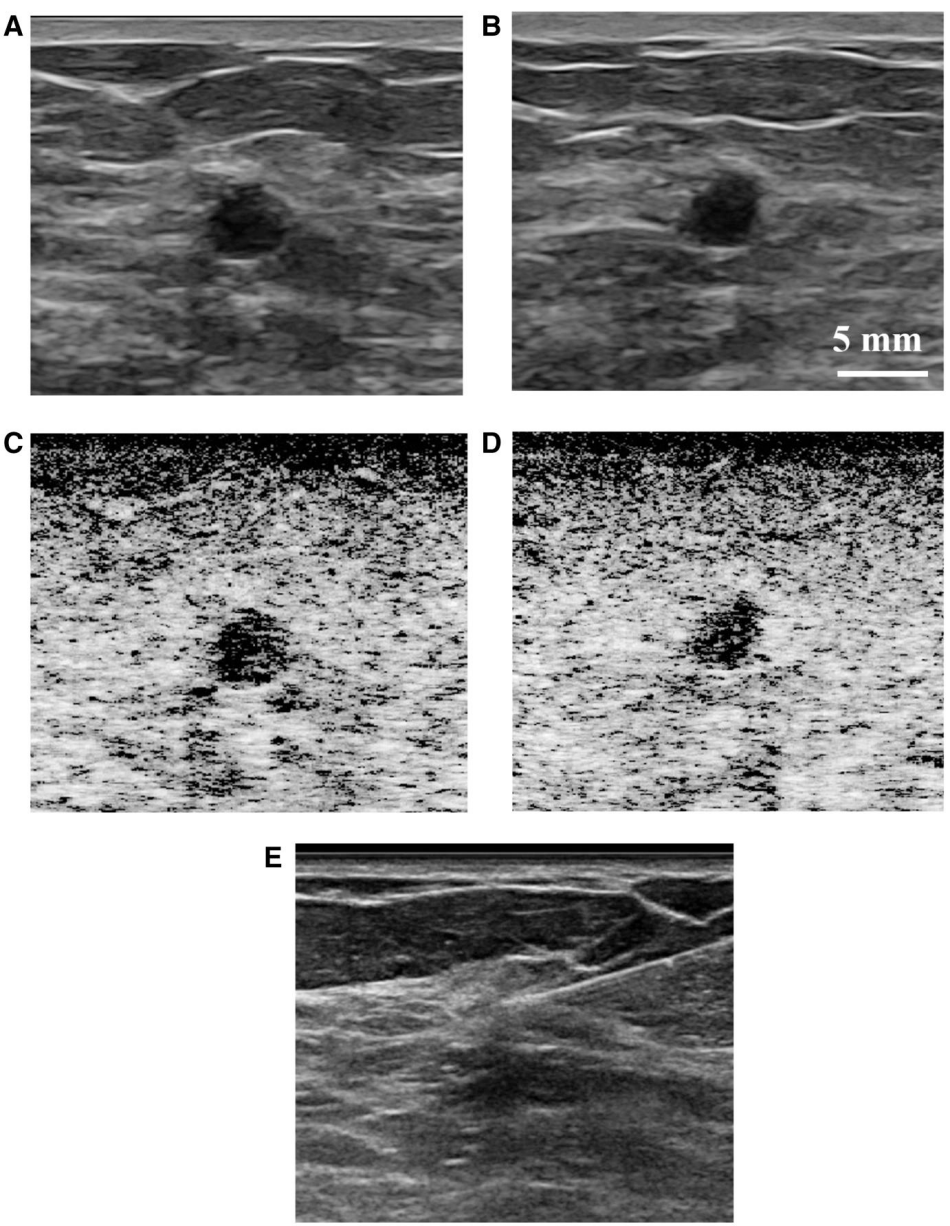
Images from a 66-year-old woman called back from screening mammogram for a left breast mass. Pairs of B-mode ultrasound static images show a 5 mm solitary circumscribed mass vs complicated cyst seen in the (A) antiradial and (B) radial projections at 1 cm depth from the ultrasound probe. (C) Antiradial and (D) radial SLSC images of the same finding show darker (or less coherent) lesion content relative to the surrounding tissue. One out of 6 board-certified radiologists characterized the finding as fluid based on B-mode ultrasound images presented in the reader study, and 5 out of 6 board-certified radiologists characterized the finding as fluid based on the SLSC images. The 1 radiologist who characterized the finding as solid with B-mode images and uncertain with SLSC images, drew regions of interest that resulted in a gCNR of 0.91 for the SLSC image, which categorizes the finding as fluid. Ultrasound-guided aspiration was performed with the (E) post-aspiration image demonstrating complete aspiration of the finding yielding benign-appearing fluid, consistent with a benign complicated cyst.

**Figure 4.**
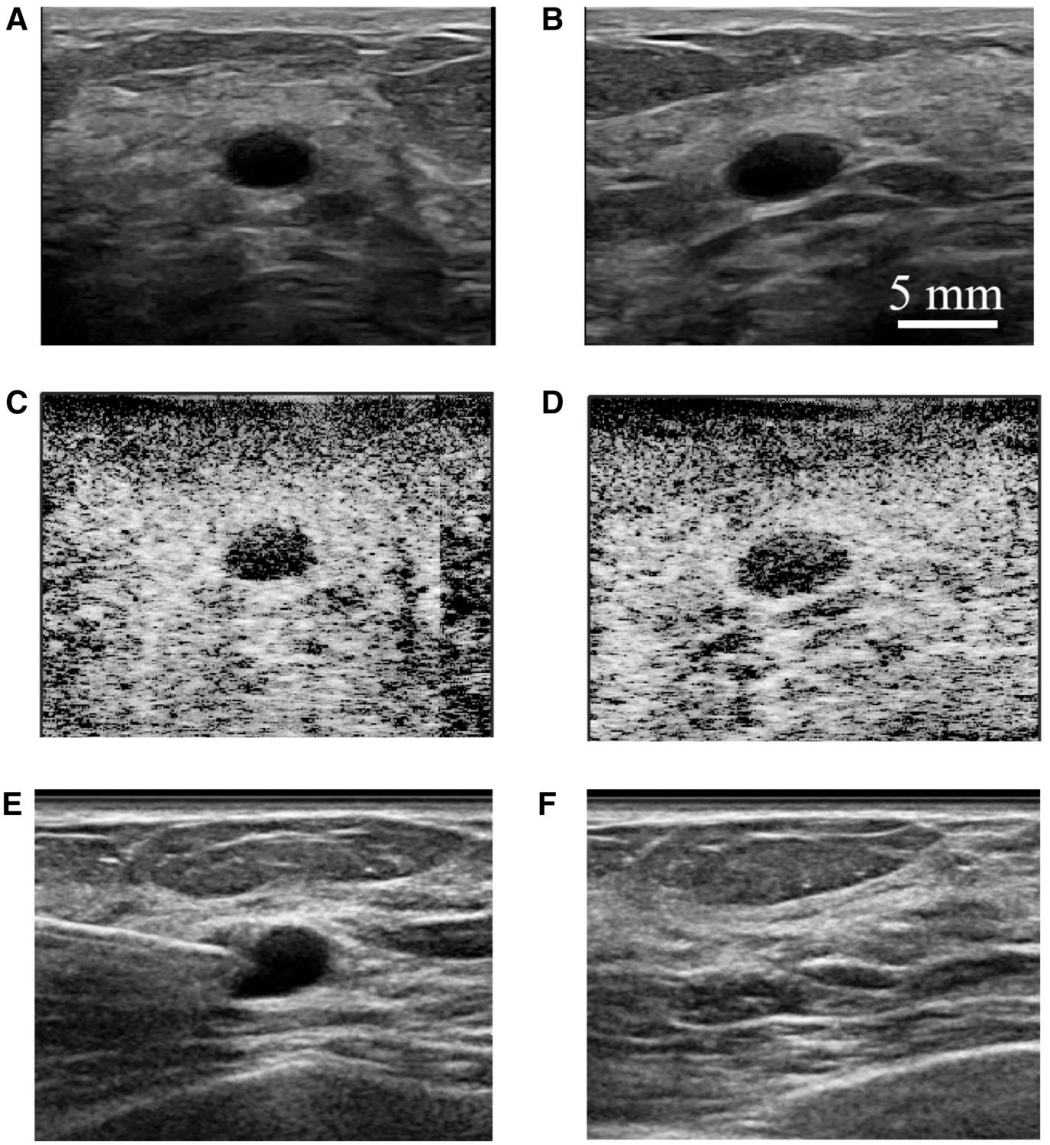
A 60-year-old woman was evaluated on follow-up imaging of a left breast sonographic mass, which had slightly increased in size compared to a prior ultrasound. Pairs of B-mode ultrasound static images show a 6 mm mass seen in the (A) antiradial and (B) radial projections at 8 mm depth from the ultrasound probe. (C) Antiradial and (D) radial SLSC images of the same finding show darker (or less coherent) lesion content relative to the surrounding tissue. Six radiologists characterized the finding as fluid based on B-mode and SLSC ultrasound images. Five of the 6 radiologists drew ROIs resulting in gCNR > 0.76 for the SLSC image, with a gCNR of 0.75 produced by 1 radiologist. Ultrasound-guided aspiration was performed with the (E) pre- and (F) post-aspiration image demonstrating complete aspiration of the finding yielding benign-appearing fluid, consistent with a benign complicated cyst.

**Figure 5.**
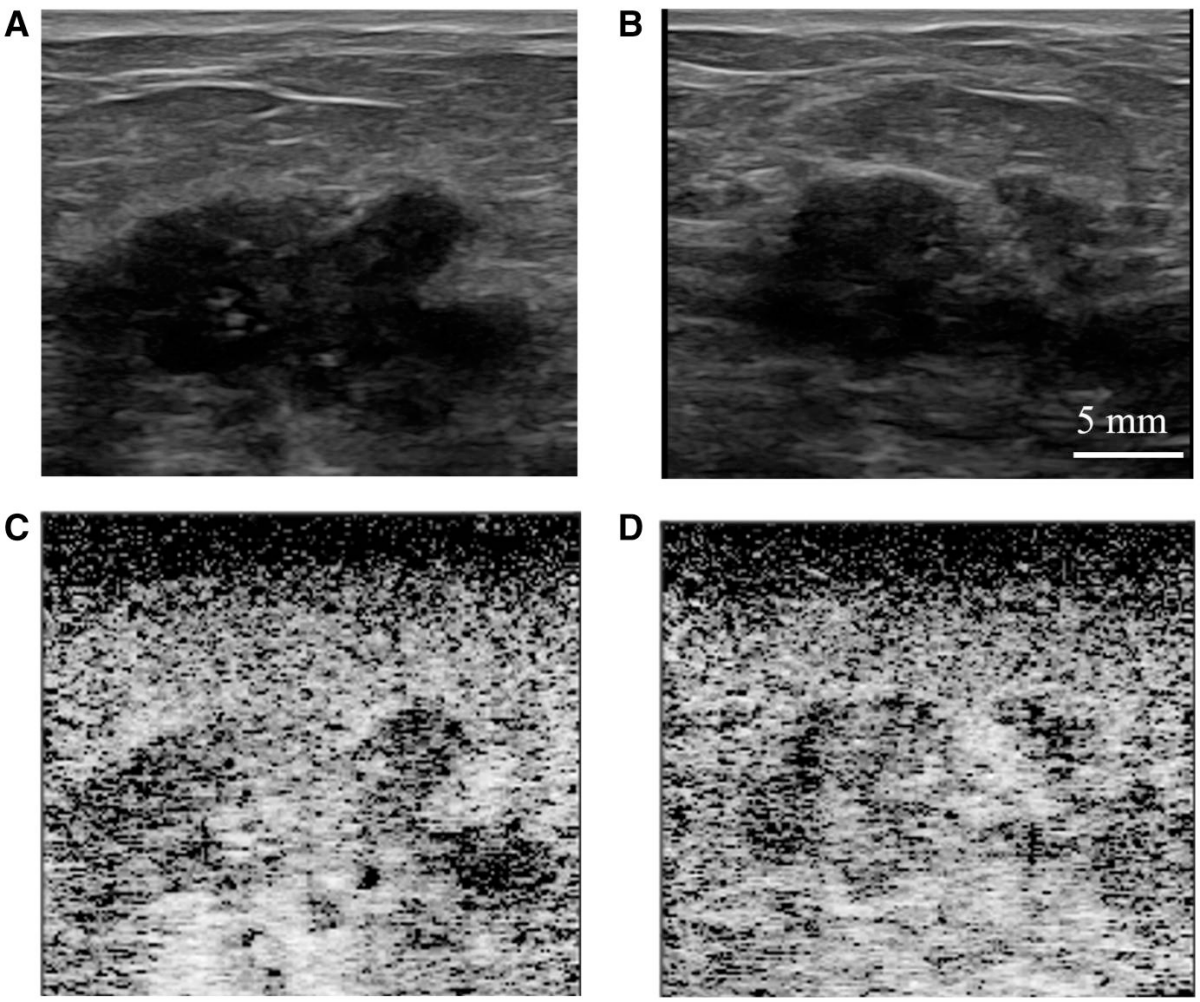
A 69-year-old woman was called back from screening mammogram for a left breast focal asymmetry. Pairs of B-mode ultrasound static images show a 3.1 cm suspicious mass seen in the (A) antiradial and (B) radial projections at 1.5 cm depth from the ultrasound transducer. (C) Antiradial and (D) radial SLSC images of the same finding show similarly coherent lesion content relative to the surrounding tissue. Five out of 6 radiologists characterized the finding as solid based on B-mode and SLSC ultrasound images, 1 radiologist characterized the finding as mixed based on B-mode and SLSC ultrasound images, and the 6 radiologists each drew regions of interest that resulted in gCNR values ranging 0.14 to 0.36 for the SLSC image, which categorizes the finding as solid. The mass was biopsied, and the pathology report revealed an invasive ductal carcinoma, moderately differentiated.

Figure 2 shows the distribution of gCNR values across various mass categories for 1 radiologist with prior experience reading SLSC images. Values closer to 1 indicate fluid content, whereas values closer to 0 indicate solid content. This distinction is more prominent with SLSC rather than B-mode images, primarily because of the lower gCNR values of solid masses in SLSC images compared to B-mode images created from the same raw data. The dashed line shows a gCNR threshold of 0.76 selected to distinguish fluid from solid masses in SLSC images. At this threshold, 8 of the 16 complicated cysts initially categorized as BI-RADS 3 and 4 were correctly identified as cysts with the gCNR metric applied to SLSC images, while 95 of the 96 solid masses were not considered cysts. Notably, no malignant masses were misclassified by gCNR. Examples highlighting the benefits of SLSC and gCNR are shown in Figures 3-5.

### Comparison of gCNR with subjective reader assessments

**Figure 6.**
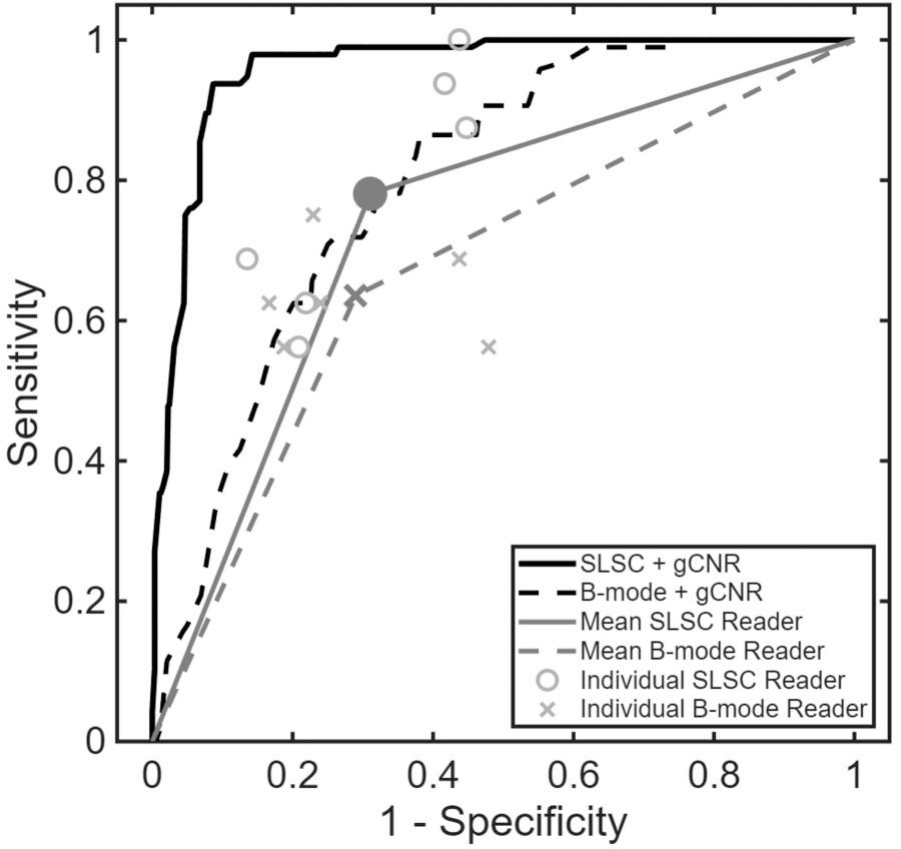
Mean ROC curves when distinguishing 16 complicated cysts from 96 solid masses by applying gCNR to B-mode (black dashed line) and SLSC (black solid line) images, showing performance with multiple gCNR thresholds. For comparison, the subjective assessment of internal contents is shown for B-mode (x) and B-mode+SLSC (o) image readings performed by each radiologist, with corresponding mean values per B-mode (**x**) or B-mode+SLSC (●) image readings. The associated ROC curves used to calculate mean lower-bound AUC values for B-mode and SLSC image readings are also shown (gray dashed and solid lines, respectively).

Sensitivity to complicated cyst classification vs 1-specificity for each reader, and the associated mean values, were plotted relative to mean ROC curves of gCNR performance (Figure 6). The ROC curves of individual readers are included as Supplementary Material. The 6 data points from the individual radiologist assessments with B-mode images reside below the mean ROC curve obtained with gCNR applied to SLSC images, with individual AUC differences ranging from 0.17 to 0.42 (*P* < .05), as reported in Table 2. Therefore, there are multiple gCNR thresholds that provide better sensitivity and specificity than subjective assessments. The mean lower-bound AUC with the subjective assessments is 0.67 (range: 0.54-0.76) with B-mode images and 0.74 (range: 0.68-0.78) with B-mode+SLSC images (Table 2). The availability of SLSC images in addition to B-mode images improved the lower-bound AUC for 2 of the 6 readers (*P* < .05). For comparison, the mean AUC is 0.80 (95% CI: 0.76, 0.84; range: 0.71 to 0.87) for distinction of complicated cysts from solid masses with gCNR applied to B-mode images. With gCNR applied to SLSC images, the mean AUC increased to 0.96 (95% CI: 0.94, 0.97; range: 0.93 to 0.98), with statistically significant improvements for the 6 radiologists (*P* < .05).

**Table 2.** AUC of complicated cyst vs solid mass classification on ultrasound in a subgroup of 112 masses, comparing subjective readings of B-mode and B-mode+short-lag spatial coherence (SLSC) images with the more objective generalized contrast-to-noise ratio (gCNR) applied to B-mode and SLSC images.

	Readings			gCNR			AUC difference between B-mode readings and gCNR applied to SLSC images
	B-mode	B-mode+ SLSC	AUC difference	B-mode	SLSC	AUC difference	
Radiologist 1	0.688	0.703	0.015 (*P* = .662)	0.818	0.972	0.154 (*P* = .004)	0.284 (*P* = 4.068e-5)
Radiologist 2	0.542	0.760	0.218 (*P* = .001)	0.836	0.965	0.130 (*P* = .003)	0.424 (*P* = 9.261e-10)
Radiologist 3	0.729	0.776	0.047 (*P* = .160)	0.870	0.977	0.107 (*P* = .002)	0.248 (*P* = 2.466e-4)
Radiologist 4	0.625	0.781	0.156 (*P* = .016)	0.792	0.943	0.152 (*P* = .001)	0.319 (*P* = 2.273e-5)
Radiologist 5	0.693	0.677	0.016 (*P* = .681)	0.793	0.968	0.176 (*P* = 3.770e-5)	0.276 (*P* = 2.223e-5)
Radiologist 6	0.760	0.714	0.046 (*P* = .353)	0.708	0.928	0.221 (*P* = 2.827e-5)	0.169 (*P* = .008)
Mean AUC ± standard deviation	0.673 ± 0.078	0.735 ± 0.043		0.803 ± 0.055	0.959 ± 0.019		
95% confidence intervals for mean AUC	(0.611, 0.722)	(0.704, 0.765)		(0.763, 0.837)	(0.944, 0.972)		

### Inter-reader agreement

The Fleiss’ *κ* values indicated fair inter-reader agreement when reading B-mode images (*κ  *=  0.40), whereas moderate inter-reader agreement was achieved when reading B-mode+SLSC images (*κ  *=  0.41), and a statistically significant moderate agreement improvement was achieved when gCNR was applied to SLSC images with the 0.76 threshold (*κ  *=  0.59, *P* < .00001), as reported in Table 3. Although inter-reader agreement can be further improved with lower gCNR thresholds, the risk of missed cancers is greater with lower thresholds. For example, at the 0.73 threshold, with the radiologist-specific ROIs, the inter-reader agreement was substantial (*κ  *= 0.70), 10-14 of the 16 complicated cysts initially categorized as BI-RADS 3 and 4 were correctly identified as cysts, and 90-93 of the 96 solid masses were correctly classified as solid, but 1-3 malignant masses per radiologist were misclassified by gCNR. This tradeoff is reported to raise awareness of potential limitations. A summary of the inter-reader ROI variability is provided in the Supplementary Material.

**Table 3.** Fleiss’ *κ* values and comparisons of inter-reader agreement.

		gCNR applied to SLSC		*κ* difference between B-mode readings and gCNR applied to SLSC images with a 0.73 threshold	*κ* difference between B-mode readings and gCNR applied to SLSC images with a 0.76 threshold
B-mode	B-mode + SLSC	0.73 threshold	0.76 threshold		
0.40	0.41	0.71	0.59	0.31 (*P* < .00001)	0.18 (*P* < .00001)

Abbreviations: gCNR = generalized contrast-to-noise ratio; SLSC = short-lag spatial coherence.

## Discussion

Ultrasound plays an important role in breast imaging but is limited by its high false positive rate.^4^ The results of prior studies indicate that fundamental SLSC imaging and gCNR have the potential to improve the diagnostic reproducibility and reader certainty of breast mass contents.^16–18^ Herein, from a cohort of 145 lesions, we evaluated the performance of gCNR as an objective metric to distinguish complicated breast cysts from solid masses in a subgroup of 112 lesions. We found that applying gCNR enabled accurate differentiation of complicated cysts from solid masses, particularly when used in the setting of SLSC imaging (ie, mean AUC of 0.96 with gCNR applied to SLSC images compared to mean lower-bound AUC of 0.67 with B-mode imaging, with *P* < .05 for the 6 readers). These findings suggest that gCNR applied to SLSC images has the potential to improve the performance of breast ultrasound by allowing for more confident complicated cyst classification, thereby reducing the need for follow-up exams, diagnostic cyst aspiration, or biopsy procedures.

Complicated cysts can sometimes be challenging to confidently diagnose due to the presence of internal debris and imaging artifacts such as acoustic clutter. SLSC imaging provides high contrast in cysts and low contrast in solid masses, therefore enabling more confident cyst classification.^15^^,^^16^^,^^19^ Improved certainty of cyst classification with breast ultrasound may be helpful in potentially reducing unnecessary ultrasound procedures and follow-up exams, thereby improving clinical resource utilization. It could also have the added benefit of reducing anxiety for patients undergoing breast ultrasound imaging evaluation for screening or diagnostic purposes.^33^

Variability in breast ultrasound performance and interpretation is recognized as an important factor that can impact the accuracy and outcomes of this modality.^15^^,^^19^^,^^34^ The results of our study showed that although SLSC imaging improved the accuracy of cyst vs solid mass classification for radiologists participating in the reader study, there was wide variation observed in the subjective assessment of internal mass contents by the radiologists. This variation highlights the benefit that gCNR can have in standardizing breast ultrasound interpretation and reducing inter-reader variation in the identification and classification of breast cysts vs solid masses. Although readers had full control over tunable parameters (eg, M in SLSC images, dynamic range of SLSC and B-mode images), which possibly increased variability, gCNR was computed on SLSC images formed with fixed parameters, further supporting the potential for standardization with gCNR.

To avoid bias and ensure standardization, the reader study was performed 2 months to 5 years after image acquisition, and the readers were not involved in the preparation of the reader study or in the analysis of the reader study results. Additionally, the same clinical history statement was provided to the readers for each case. Although clinical history is typically known and plays a role in image interpretation and assessments in realistic clinical settings, a standardized clinical history allowed for the assessment of SLSC-related improvements without the potential confounder of differences in provided clinical histories.

While artificial intelligence (AI) solutions have been developed for breast ultrasound, aiming to differentiate benign from malignant breast lesions,^35^ SLSC directly exploits the underlying physics of tissue scattering. Future work could potentially integrate coherence-based imaging with AI-based tools to further enhance diagnostic performance.

Our study had limitations, with one being the static presentation of sonographic findings to readers, which likely introduced bias. Additionally, this study was performed at a single academic institution using a single ultrasound scanner. Generalizability across different scanners, medical practice sites, and patient cohorts was not demonstrated. Moreover, gCNR was not tested or implemented for cyst classification using different ultrasound vendors or other investigators in other practice settings. Another limitation is that superficial breast masses may not be suitable for SLSC categorization, because it is difficult to focus ultrasound in the near-field region,^17^ resulting in a 4/175 (2%) SLSC imaging failure rate for our study. Barriers to clinical adoption include the time needed to train, gain technological familiarity, and manually draw ROIs. Furthermore, our prototype SLSC code and our custom post-processing software are not commercially available. However, the technology used in our study can be independently implemented and confirmed using any ultrasound scanner that provides raw ultrasound channel data. SLSC code^20^ can then be applied, followed by gCNR measurements.^22^^,^^23^ Potential next steps to overcome the remaining barriers include utilizing automated ROIs and creating customizable training programs that allow radiologists to see multiple case examples and efficiently gain familiarity. Although our reported AUC values exclude complex cystic and solid masses, the application of SLSC and gCNR to cases where it may otherwise be challenging to differentiate complex cystic and solid masses from complicated cysts can be addressed in future research.

In conclusion, our study results indicate that SLSC imaging, combined with objective gCNR criteria, can improve accuracy and reader agreement when classifying complicated cysts vs solid masses and therefore has the potential to reduce unnecessary downstream ultrasound procedures.

## Supplementary Material

umaf037_Supplementary_Data

## Data Availability

The data underlying this article cannot be shared publicly at this time, to ensure compliance with the consent of individuals who participated in the study. Data are available for research purposes upon reasonable request to the corresponding author, with institutional approval required.
