# Supplementary material for: Generalized contrast-to-noise ratio applied to short-lag spatial coherence ultrasound differentiates breast cysts from solid masses
Source: Radiol Adv. 2025 Oct 24;2(6):umaf037. doi: 10.1093/radadv/umaf037 (PMC12709606; doi:10.1093/radadv/umaf037)
Supplement: umaf037_Supplementary_Data [file umaf037_supplementary_data.zip › SupplementaryMaterial.pdf]

Generalized contrast-to-noise ratio applied to short-lag spatial coherence ultrasound differentiates breast cysts from solid masses
(Supplementary Material)

Arunima Sharma, PhD[1*], Eniola T. Oluyemi, MD, MPH[2*], Madhavi Tripathi, PhD[1], Emily B. Ambinder, MD, MS[2], Lisa A. Mullen, MD[2], Babita Panigrahi, MD[2], Joanna Rossi, MD, MPH[2], Nethra Venkatayogi, BS[1], Kelly S. Myers, MD[2**], and Muyinatu A. Lediju Bell, PhD[1,2**]

[1]Johns Hopkins University, Baltimore, MD 21218
[2]Johns Hopkins Medicine, Baltimore, MD 21287
[*]Equal contribution; **Co-senior authors

1 Reader Study Training Process

Prior to completing Tasks 1 and 2 of the reader study, training consisted of a presentation that provided an overview of the technology, including one example each of a simple cyst, complicated cyst, benign solid mass, malignant solid mass, and complex cystic and solid mass (none were included in the actual study), and key features to consider when classifying masses with SLSC imaging. The key takeaways were (1) dark regions = low coherence = fluid, (2) white regions = high coherence = solid, (3) primarily low coherence should be classified as fluid, (4) primarily high coherence should be classified as solid, (5) a mixture of low and high coherence should be classified as mixed, and (6) anything else should be categorized as uncertain. An optional overlay feature was also introduced to display SLSC images in color rather than grayscale [1]. Following the presentation, each participant completed a tutorial with two example masses seen during training, using the graphical user interface shown in Fig. S1. The entire training lasted approximately 15 minutes.

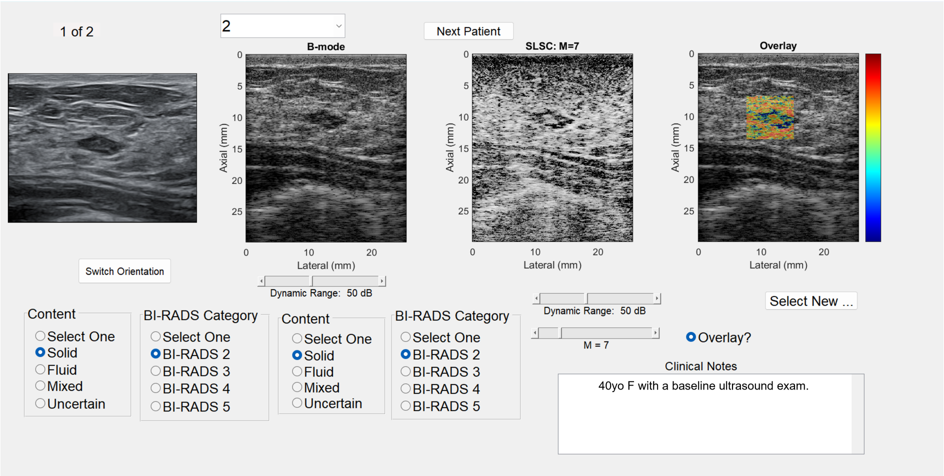

Figure S1: Visualization of the graphical user interface

2 Inter-Reader ROI Variability

To complete Task 3, the same six readers, blinded to final pathology, independently drew two regions of interest (ROIs) that were used to calculate gCNR. The rules for drawing the ROI are described in the manuscript. Specifically, the ROIs within masses and surrounding tissue were manually selected from B-mode images. The mass ROI was an elliptical area of size, aspect ratio, and position selected by the readers. The corresponding tissue ROI was the same size and shape as the mass ROI, placed in a reader-selected position. The guidelines provided to the radiologists were to (1) draw the mass ROI within the mass, excluding the mass boundary, and (2) place the associated tissue ROI at the same depth as the mass ROI. Radiologists were also allowed to judge the best possible tissue ROI, depending on the surrounding parenchyma. Examples of ROIs chosen by the radiologists are shown in Fig. S2 for a single lesion (i.e., a 17 mm ductal carcinoma in situ). Table S1 summarizes the ROI sizes obtained with the 145 masses included in Task 3, including a summary of the mean, standard deviation, minimum, and maximum ROI areas per reader.

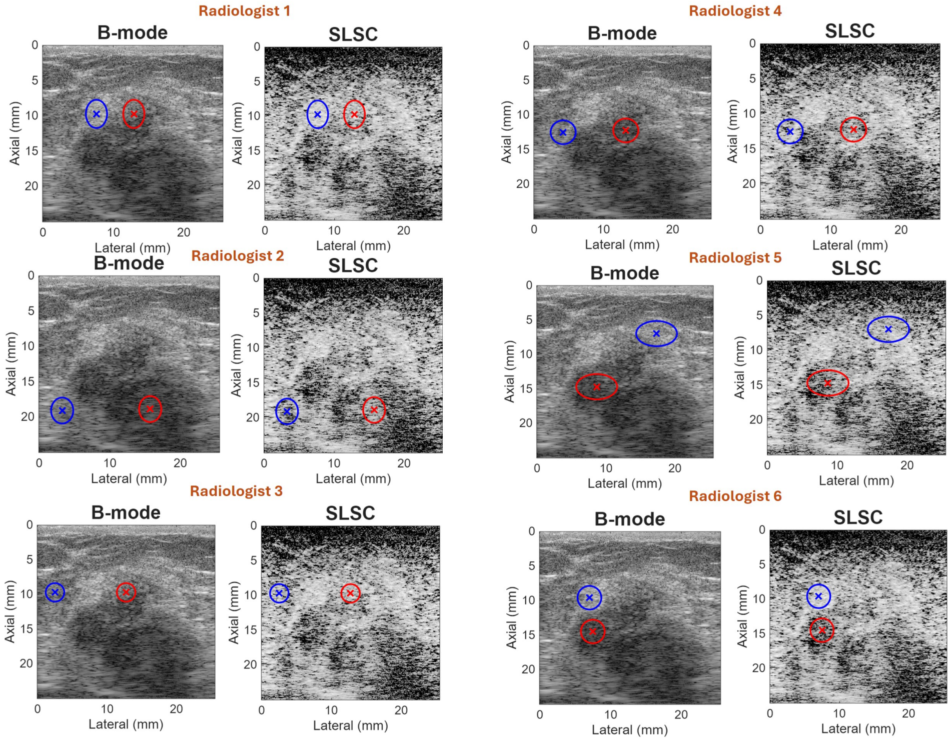

Figure S2: Example of ROIs drawn by six radiologists during Task 3 of the reader study. Mass and tissue ROIs are shown in red and blue, respectively. Radiologists only had access to B-mode images when drawing the ROIs, and the corresponding ROIs were applied to SLSC images created from the same raw data.

Table S1: Summary of ROI size variations

	Mean	Standard Deviation	Minimum	Maximum
**ROI Area (mm^2)**				
Radiologist 1	12.8	13.6	0.4	100.5
Radiologist 2	13.3	11.6	1.0	62.8
Radiologist 3	13.3	12.6	0.8	83.5
Radiologist 4	13.0	12.6	0.8	100.5
Radiologist 5	8.8	8.1	1.3	62.8
Radiologist 6	9.2	10.9	0.8	100.5

3 Calculating gCNR from ROIs

For each mass, the same mass and tissue ROIs drawn by each reader on each B-mode image were implemented when calculating the gCNR of B-mode and SLSC images, as follows [2, 3, 4]:

$$\text{gCNR} = 1 - \sum_{j=1}^{\mathcal{N}} \min\{h_{mass}(x_j), h_{tissue}(x_j)\} \quad (1)$$

where $\mathcal{N}$ bins centered at $\{x_1, x_2 ... x_{\mathcal{N}}\}$ were defined to derive histograms h_{mass} and h_{tissue}. The calculated gCNR values resulting from the example ROIs shown in Fig. S2 are reported in Table S2. The size of the ROIs corresponding to these gCNR values are also reported. Variations in ROI selections among readers contribute to gCNR variability for the same mass, which contributes to different classifications of fluid vs. solid based on the chosen gCNR threshold.

Table S2: gCNR values (and corresponding ROI areas) measured using the radiologist-drawn ROIs shown in Figure S2

	B mode gCNR	SLSC gCNR	ROI Area (mm^2)
Radiologist 1	0.680	0.394	9.4
Radiologist 2	0.670	0.151	9.0
Radiologist 3	0.168	0.101	5.6
Radiologist 4	0.747	0.346	9.7
Radiologist 5	0.831	0.387	17.0
Radiologist 6	0.906	0.760	9.0

4 ROC Curves of Individual Readers

Fig. S3 shows the ROC curves of gCNR performance with multiple thresholds, relative to corresponding data points showing sensitivity to complicated cyst classification vs. 1-specificity for the same radiologists. Six data points from the subjective radiologist assessments with B-mode images reside below the corresponding mean ROC curves obtained with gCNR applied to SLSC images. Therefore,

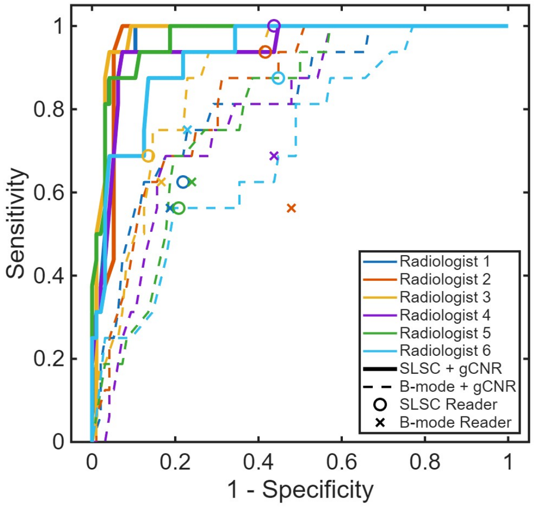

Figure S3: ROC curves when distinguishing 16 complicated cysts from 96 solid masses by applying gCNR to B-mode (dashed lines) and SLSC (solid lines) images, showing performance with multiple gCNR thresholds. For comparison, the subjective assessment of internal contents is shown for B-mode (x) and B-mode + SLSC (o) image readings.

there are multiple gCNR thresholds that provide better sensitivity and specificity than subjective assessments. One data point from the subjective assessment with B-mode images (Radiologist 6) resides above the corresponding ROC curve describing gCNR performance on B-mode images, hence subjective readings are better in this case. One data point from the subjective assessment with B-mode combined with SLSC images (Radiologist 4) shares similar sensitivity and specificity with the corresponding SLSC + gCNR ROC curve, which means that there is one gCNR threshold that is as good as the subjective reading process. Overall, the application of gCNR to SLSC images with multiple possible thresholds consistently outperformed gCNR applied to B-mode images and subjective readings of B-mode and B-mode+SLSC images.

References

[1] A. Wiacek, E. Falomo, K. Myers, O. M. H. Rindal, K. Fabrega-Foster, S. Harvey, and M. A. L. Bell, "Clinical feasibility of coherence-based beamforming to distinguish solid from fluid hypoechoic breast masses," in *Proceedings of the IEEE International Ultrasonics Symposium (IUS)*, pp. 1–4, IEEE, 2018.

[2] A. Rodriguez-Molares, O. M. H. Rindal, J. D'hooge, S.-E. Måsøy, A. Austeng, M. A. L. Bell, and H. Torp, "The generalized contrast-to-noise ratio: a formal definition for lesion detectability," *IEEE Transactions on Ultrasonics, Ferroelectrics, and Frequency Control*, vol. 67, no. 4, pp. 745–759, 2020.

[3] M. R. Gubbi, E. A. Gonzalez, and M. A. L. Bell, "Theoretical framework to predict generalized contrast-to-noise ratios of photoacoustic images with applications to computer vision," *IEEE Transactions on Ultrasonics, Ferroelectrics, and Frequency Control*, vol. 69, no. 6, pp. 2098–2114, 2022.

[4] A. Sharma, E. Oluyemi, K. Myers, E. Ambinder, and M. A. L. Bell, "Spatial coherence approaches to distinguish suspicious mass contents in fundamental and harmonic breast ultrasound images," *IEEE Transactions on Ultrasonics, Ferroelectrics, and Frequency Control*, vol. 71, no. 1, pp. 70–84, 2024.
